# The role of geographic bias in knowledge diffusion: a systematic review and narrative synthesis

**DOI:** 10.1186/s41073-019-0088-0

**Published:** 2020-01-15

**Authors:** Mark Skopec, Hamdi Issa, Julie Reed, Matthew Harris

**Affiliations:** 1 Department of Primary Care and Public Health, Imperial College London, Reynolds Building, St Dunstan’s Road, London, W6 8RP UK; 2 Institute of Global Health Innovation, St Mary’s Hospital, Praed Street, London, W2 INY UK; 3 NIHR CLAHRC North West London, 369 Fulham Road, London, SW10 9NH UK

**Keywords:** Geographic bias, Randomized controlled trials, Systematic review, Narrative synthesis

## Abstract

**Background:**

Descriptive studies examining publication rates and citation counts demonstrate a geographic skew toward high-income countries (HIC), and research from low- or middle-income countries (LMICs) is generally underrepresented. This has been suggested to be due in part to reviewers’ and editors’ preference toward HIC sources; however, in the absence of controlled studies, it is impossible to assert whether there is bias or whether variations in the quality or relevance of the articles being reviewed explains the geographic divide. This study synthesizes the evidence from randomized and controlled studies that explore geographic bias in the peer review process.

**Methods:**

A systematic review was conducted to identify research studies that explicitly explore the role of geographic bias in the assessment of the quality of research articles. Only randomized and controlled studies were included in the review. Five databases were searched to locate relevant articles. A narrative synthesis of included articles was performed to identify common findings.

**Results:**

The systematic literature search yielded 3501 titles from which 12 full texts were reviewed, and a further eight were identified through searching reference lists of the full texts. Of these articles, only three were randomized and controlled studies that examined variants of geographic bias. One study found that abstracts attributed to HIC sources elicited a higher review score regarding relevance of the research and likelihood to recommend the research to a colleague, than did abstracts attributed to LIC sources. Another study found that the predicted odds of acceptance for a submission to a computer science conference were statistically significantly higher for submissions from a “Top University.” Two of the studies showed the presence of geographic bias between articles from “high” or “low” prestige institutions.

**Conclusions:**

Two of the three included studies identified that geographic bias in some form was impacting on peer review; however, further robust, experimental evidence is needed to adequately inform practice surrounding this topic. Reviewers and researchers should nonetheless be aware of whether author and institutional characteristics are interfering in their judgement of research.

## Background

Descriptive studies observe a noticeable skew of published research toward high-income countries (HICs) and institutions of significant scientific repute [1–3]. Indeed, a global North-South research gap still exists, with most scientific contributions originating from the U.S, the UK, Canada, and Australia [4], and a remarkably high spatial concentration of scientific activity in Europe [5]. North America and Europe receive 42.3% and 35.3% of the world’s citations, respectively, while the total contribution of the world’s citations from Africa, South America, and Oceania is lower than 5% [6]. Citation counts increase exponentially with increasing gross domestic product (GDP) [7].

Although this may be due to scientific capability, research production, and the quality of research, it is possible that research from low-and-middle-income country (LMIC) contexts is being discounted prematurely and unfairly, due to a bias against the country from which the research originates. Many argue that a significant portion of the world is being overlooked when it comes to scientific contributions, [5–9]. Bias may occur at any stage in the review and publication process [10]. Heuristics, or mental shortcuts, offer a possible explanation for this skew of scientific research [11, 12]. Research articles possess intrinsic and extrinsic cues as to their quality [13]. Intrinsic cues are attributes that cannot be changed, such as the research methods [13]. The quality may be judged, for example, on adherence to the stated methods, and the strength of the evidence in the research, i.e., its internal validity. Extrinsic cues are informal stimuli that may be used, even unwittingly, to make judgments about a given research article, most notably as it relates to its quality [14].

Country of origin (COO) effects, for example, are a specific type of extrinsic cue where the country source influences a consumer’s perception of the product [15, 16]. COO effects can explain the association consumers make between HICs and high-quality products. Consumer preference is positively correlated with the degree of economic development of the source country [17]. In such scenarios, country development status, an extrinsic cue, is used to infer product quality. HICs evoke an image of technologically advanced societies, and in the consumer’s mind, this technological advancement is necessary to produce high-quality goods. Conversely, certain consumers associate products from LMICs with poorer quality, increased risk of bad performance, and dissatisfaction, due to the lesser degree of economic development [14]. If research articles are considered a product, albeit an intellectual one, it is possible that a COO effect may be equally elicited in research review at any stage in the publication process.

Peters and Ceci’s experiment to test the reliability of the peer review process [18] was the first to highlight this issue. By altering the authorship of 12 research papers to fictional or unknown institutions they found that only one of the 12 papers resubmitted to the same journals that had previously published them a few years earlier was accepted for publication [18]. Considering Peters and Ceci’s findings, coupled with the COO effects outlined above, it is conceivable that a similar phenomenon may be observable in the evaluation of research from LMICs as well. Just as the source of a product influences the consumer’s choice to purchase it, the geographic origin of a scientific manuscript may bias a reviewer or a reader’s opinion of the research. Extrinsic cues, such as COO (equating LMICs with low-quality research) may guide the decision-making process.

Studies using implicit association test methodology have found that unconscious bias toward research from LMICs is prevalent [19]. Recently, McGillivray et al. found that articles submitted to Nature journals are less likely to progress through the publication process if from low-prestige institutions [20]. Although studies examining citation counts show that publication and citation frequency is skewed toward HICs [1–3, 21], these retrospective, descriptive studies cannot definitively address [22, 23] whether this is due to geographic bias, because these designs do not shed light on whether consumers of research (whether editors, peer reviewers, or readers) are biased by the geographic origin of the research, as opposed to, for example, considering the relevance or quality of the research. Randomized controlled trials (RCTs) are the best way to determine whether the external cue of COO is influencing how reviewers rate research articles [24]. RCTs of the role of geographic bias could inform policy on best practice in peer review and beyond. We describe a systematic review to identify RCTs that specifically examine geographic bias in the assessment of the quality of research articles to determine its full extent in the knowledge diffusion and publication process.

## Methods

### Search strategy

A systematic search of bibliographic databases was performed during June 2018. No time filter was applied for the search, to not restrict the already limited research available on this topic. Databases included MEDLINE, Embase, Global Health, and PsycInfo. Health Management Information Consortium (HMIC) was searched as a source of gray literature. Additional articles were identified through hand-searching the reference lists of screened full-text articles. Authors of included full-text articles were contacted and asked about their knowledge of further relevant studies.

Search terms were identified using the SPIDER tool [25]. This tool was chosen as it has been found to have greater specificity than comparable search tools (such as PICO) in qualitative evidence synthesis [26]. Using the tool as a framework, we devised search terms for each of the different categories. The “Sample” category included terms such as “Periodicals as Topic/”, “Publications/”, and “peer review.” The “Phenomenon of Interest” included terms such as “Bias” or “Prejudice.” The “Design” category would have included terms such as “RCT” or “Randomized Controlled Trial,” but it was felt that including this term in the search strategy could serve to further limit already scarce evidence of the phenomenon we were seeking to investigate. The “Evaluation” category included terms such as “Observer variation,” “implicit,” and “explicit.” Finally, the “Research Type” category would have focused on quantitative research, but, as with the “Design” category, it was decided to omit search terms in this category. Table 1 lists search terms used for each source. The search strategies for each source can be made available upon request.

**Table 1:**
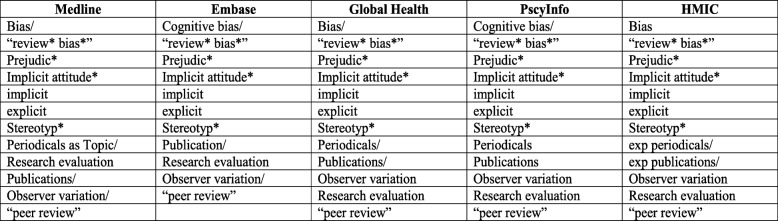
Combination of key words and MeSH terms used for to search databases

MeSH terms are followed by “/”. Keywords are in quotation marks. Asterisks (*) denote truncation of keywords

### Inclusion criteria

One reviewer (MS) screened retrieved titles. Two authors (MS and MH) then independently reviewed abstracts for inclusion. A consensus was reached surrounding subsequent inclusion of reviewed abstracts. Full-text articles were reviewed by MS and MH jointly. Decision to include full texts was reached by consensus between both reviewers. Both authors assessed the quality of reviewed articles. Articles were included if they were peer reviewed publications of intervention studies where the primary outcome was a quantitative research review score (relative risks (RR) or odds ratios (OR) of acceptance) assessing the role of nationality, geographic, or institutional affiliation bias among reviewers or editors of periodical journals or other scientific publications. Secondary outcomes considered for inclusion were the categorical classification of manuscripts (recommendation for review and resubmission, acceptance for publications or outright rejection). Articles published in languages other than English were considered if titles and/or abstracts seemed relevant. In these cases, authors were contacted to obtain English-language transcripts, if possible.

Only randomized, controlled intervention studies were included to ascertain the individual-level effect of geographic bias. Articles were not considered for inclusion if they did not specifically examine an aspect of geographic bias, such as the role of institutional affiliation, COO, or a variant thereof or were non-randomized, non-intervention, or descriptive studies (such as bibliometric analyses of citation counts and citation tracking, review articles, editorials, or “letters to the editor.”) because these retrospective or descriptive studies cannot offer reliable evidence regarding individual-level biases [22, 23]. We included studies that explored any aspect of geographic bias, i.e., local, regional, national, or international.

### Data abstraction

Search results were merged using reference management software (Zotero 5.0.53) to remove duplicate records. Records were exported to a spreadsheet for screening. If deemed relevant to the scope of the review according to the inclusion criteria, or if the scope was unclear from the title, abstracts were reviewed. After identification of relevant abstracts, full-text articles were reviewed. The same screening strategy was employed for articles identified through hand-searching. Where appropriate, investigators were contacted to clarify study eligibility and to determine if they were aware of similar studies. If concerns and questions about inclusion persisted upon completion of the full-text review, these were discussed within the research team.

### Data analysis

Our familiarity with the subject matter led us to anticipate that the outcome measures of included studies would be too heterogeneous to conduct a comparison using a robust meta-analysis. As such, we determined a priori that a narrative synthesis would be the most appropriate method to compare eventual findings.

## Results

### Study selection and characteristics

**Fig. 1:**
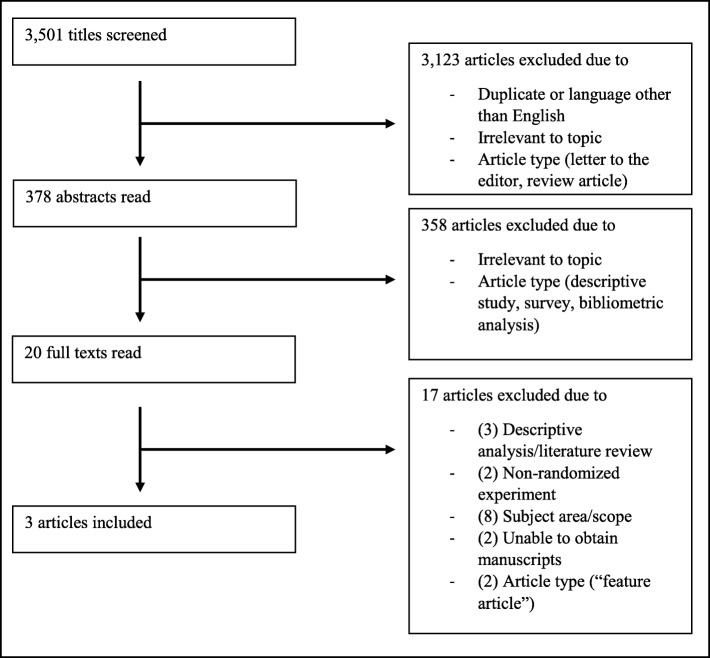
Flowchart detailing the study selection process

The systematic literature search yielded 3501 titles. Upon removing duplicates, 3255 titles were screened. After screening of titles, 378 abstracts were reviewed. From these abstracts, 12 full texts were reviewed for inclusion, and a further eight were identified through searching reference lists of the full texts. Of these 20 articles, three were found to meet inclusion criteria for narrative synthesis. The three corresponding authors were contacted, but no further studies were retrieved through these means. A flowchart outlining the study selection process can be found in Fig. 1.

One study assessed the within-individual variation in the evaluation of a research abstract when the COO of the abstract is changed from HIC to low-income country (LIC) [27]. Two further studies investigated other dimensions of geographic bias, but still met overall criteria for inclusion. One sought to investigate three forms of bias in detail: the Matilda effect (in which papers from male authors are evaluated more favorably), the Matthew effect (in which already-famous researchers receive most of the recognition for newly published work), and the biases resulting from the fame or the prestige or ranking of the author’s institutions [28]. Notably, within this particular study, only the third objective is of relevance to this review. The third study investigated if articles published in “high-prestige” journals (as measured by journal impact factor (IF)) elicited a more positive response from the reviewers than did articles published in “low-prestige” journals [29]. As the journals investigated were the *New England Journal of Medicine* (NEJM, a high IF journal), and the *Southern Medical Journal* (SMJ, a low IF journal), both containing an explicit geographic association in their names, we included this study in our analysis.

Themes and relationships within the data were explored, compared, and discussed. A detailed investigation of sources of variability and heterogeneity between the included studies was undertaken. Validity of studies was assessed using the risk of bias assessment [30].

### Characteristics of study methodology

A summary table of the characteristics of the included studies can be seen in Table 2. Each of the characteristics is discussed in more detail below.

**Table 2:** Summary characteristics of included studies

Title	Author and year	Journal	Study question(s)	Sample size	Study design	Intervention	Outcome measures	Results
Do physicians judge a study by its cover? An investigation of journal attribution bias	Christakis, 2000	Journal of Clinical Epidemiology	Does attribution of an article to a "high-prestige" journal versus a "low-prestige" journal affect readers' impressions of the quality of the article, and does formal training in epidemiology and biostatistics mitigate these effects?	264 physicians who listed internal medicine as their primary specialty recruited from the American Medical Association’s master list of licensed physicians.	Randomized, single-blind. It is unclear from the article how randomization was achieved.	Participants were asked to read an article and abstract from either the SMJ or the NEJM. They were given the abstracts or articles either attributed or unattributed. After each article or abstract, respondents were asked to rate the quality of the study, the appropriateness of the methodology employed, the significance of the findings and its likely effects on their practice. Ratings were on a Likert scale, and responses were used to generate an aggregate ‘Impression Score’ ranging from 5-25.	Difference in ‘Impression Score’ given by reviewers who read either correctly attributed abstracts or articles or unattributed abstracts or articles.	The predicted odds for review score prediction for “Top universities” are 1.58 [95% CI (1.09–2.29]. The predicted odds for review score prediction for “Paper from the U.S.” are 1.01 [95% CI (0.66–1.55)]. The predicted odds for review score prediction for “Same country as reviewer” are 1.15 [95% CI (0.71–1.86)].
Explicit bias toward high-income country research: a randomized, blinded, crossover experiment of English clinicians	Harris, 2017	Health Affairs	Assessed the within-individual change in evaluation of research abstracts when the source is experimentally altered - in this case, between high- and low-income countries.	347 clinicians, of any speciality, living and practicing in England.	Randomized, controlled, blinded crossover experiment. The survey platform carried out simple randomization in real-time while respondents entered the survey.	Participants rated the same abstracts on two separate occasions, one month apart, with the source of these abstracts changing, without their knowledge, between high- and low-income countries. Participants were asked to rate the abstracts based on strength of evidence, relevance to their practice, and likelihood to recommend the paper to a colleague. Scores were assigned in each of these categories on a scale of 0–100.	Difference in review scores between the two rounds of reviewing, therefore comparing review scores from HIC abstracts to review scores from LIC abstracts.	Overall mean difference in rating of strength between abstracts from HIC and LIC source was 1.35 [95% CI (− 0.06–2.76)]. Overall mean difference in rating of relevance and likelihood of recommendation to a peer between abstracts HIC and LIC source was 4.50 [95% CI (3.16–5.83)] and 3.05 [95% CI (1.77–4.33)], respectively.
Reviewer bias in single- versus double-blind peer review	Tomkins, 2017	Proceedings of the National Academy of Sciences	Investigated bias resulting from the fame or quality of the authors’ institution(s).	1,957 review committee members at the Web Search and Data Mining (WSDM 2017) conference.	Randomized, double- and single-blind. The authors do not specify how reviewers were randomized into their respective groups.	Four committee members reviewed each paper. Two of these four reviewers are given access to author information (single-blind); the other two are not (double-blind). Reviewer behavior is studied in two settings: reviewing papers and also a preliminary "bidding" stage in which reviewers express interest in papers to review.	A “Blinded paper quality score” (bpqs, the average quality score of the double-blind reviews for that paper) is used as a proxy measure for the intrinsic quality of a paper. This is used to calculate the odds of acceptance among single- versus double-blind reviewers.	The predicted odds for review score prediction for “Top universities” are 1.58 [95% CI (1.09–2.29]. The predicted odds for review score prediction for “Paper from the U.S.” are 1.01 [95% CI (0.66–1.55)]. The predicted odds for review score prediction for “Same country as reviewer” are 1.15 [95% CI (0.71–1.86)].

#### Trial design

Each study included in this review used a different trial design. Harris et al. used a cross-over design, whereby each subject served as their own control [27]. The intervention involved asking clinicians to read and rate four different, previously published abstracts, fictionally attributed to either of two HIC institutions, or two LIC institutions, on two separate occasions, 4 weeks apart. One abstract was for a randomized trial of directly observed treatment, short course (DOTS) for tuberculosis (TB) treatment, one compared human immunodeficiency virus (HIV) services in maternal and child health, one was for a randomized trial for the cholesterol-lowering drug rosuvastatin, and one was a cross-sectional trial of the drug methadone in the treatment of drug addicts [27]. The author affiliations were switched between each review, so that abstracts initially attributed to HIC sources were attributed to LIC sources during the second wave of review, and vice versa.

Tomkins et al used a parallel trial design. Authors randomly assigned reviewers of an annual computer science conference to either the Single-Blind Program Committee (SBPC) or the Double-Blind Program Committee (DBPC). SBPC reviewers had access to author information, whereas DBPC reviewers did not. SBPC and DBPC members conducted their reviews simultaneously, and a predicted odds of acceptance for a list of seven covariates was generated [28]. Christakis et al. used a factorial design of all different permutations of a questionnaire for both journals and attribution status. 2^2^ × 2^2^, or 16, different questionnaires were generated and randomly distributed to participants [29]. Participants were sent either an article, or an abstract, either correctly attributed to the NEJM or the SMJ, fictionally attributed to the NEJM or the SMJ, or unattributed altogether. The first article concerned a treatment of diabetic gastroparesis, the second was a cost analysis of kinetic therapy in preventing complications of stroke, the third was a randomized trial of surgery as a treatment for metastases to the brain, and the fourth examined nephrotoxicity following treatment with angiotensin-converting enzyme (ACE) inhibitors and nonsteroidal anti-inflammatory drug (NSAID) therapy [29]. Reviewers were then asked to rate the abstracts/articles in five categories on a Likert scale from 1 to 5. This generated an aggregate review score between 5 and 25 for each abstract/article.

#### Study population

Harris et al [27] targeted English clinicians through a Qualtrics survey platform, which consists of a curated list of individuals interested in participating in research. At baseline, 551 completed surveys were obtained. Of those, 347 (63.0%) clinicians also completed the second wave of surveys. Tomkins [28] selected participants from the program committee for the Web Science and Data Mining (WSDM) 2017 conference. A total of 983 reviewers were allocated to the SBPC, and 974 to the DBPC. These reviewers evaluated a total of 500 submissions. Christakis [29] identified subjects from the American Medical Association’s (AMA) master list of licensed physicians in the U.S, and from the master list of internists who had completed the Robert Wood Johnson (RWJ) Clinical Scholars program. A total of 399 participants were found to be eligible to receive a questionnaire. In total, 264 of 399 questionnaires (66%) were returned and analyzed by the authors.

#### Randomization

All studies were randomized. Harris [27] employed simple randomization, which occurred in real time through the Qualtrics survey platform, so that participants would be unaware that randomization had taken place. The other two articles included did not specify how randomization was performed [28, 29].

#### Blinding

All three studies included a “white lie” concerning the purpose of the study. Harris [27] and Christakis [29] were deliberate in their descriptions (describing the survey as a “speed-reading survey” to “enhance anchoring and fast-thinking,” (Harris) or citing an “[examination] of how physicians use information from the medical literature” (Christakis)), thus reducing the possibility of eliciting the types of behaviors they were seeking to investigate. Whereas Tomkins [28] noted in their call for papers that was sent to authors that “WSDM 2017 will use a combination of single-blind and double-blind review,” they did not mention how or if Program Committee (PC) members were notified of this change.

#### Outcome measures

Harris [27] asked participants to rate the abstracts in the categories of strength of evidence, relevance to the reader, and likelihood of recommendation to a peer. Responses were on a scale of 0–100, with 0 being not at all strong, relevant, or likely to recommend, and 100 being very strong, relevant, or likely to recommend. Mean scores and 95% confidence intervals (CIs), as well as mean difference in scores between the first and the second review were reported. The overall mean within-individual difference in rating of strength of evidence between abstracts from HIC and LIC source was 1.35 [95% CI (− 0.06–2.76)]. The rating of relevance and likelihood of recommendation to a peer between abstracts from HIC and LIC source was 4.50 [95% CI (3.16–5.83)] and 3.05 [95% CI (1.77–4.33)], respectively.

Tomkins [28] invited reviewers to rate each paper and allocate a review score. Reviewers also entered a “rank” for the paper. Reviewers then completed a textual review of the submission. The authors then conducted a regression analysis to calculate the predicted OR that a single-blind reviewer gives a positive (accept) score to a paper. Seven covariates were investigated which could modify the predicted odds. Only three (whether the single most common country among the paper’s authors was the U.S., whether the reviewer was from the same country as the first author, and whether the paper originated from one of the top 50 global computer science universities) were relevant to the purpose of this review. The predicted odds for review score prediction for “Top universities” were 1.58 [95% CI (1.09–2.29]. The predicted odds for review score prediction for “Paper from the U.S.” was 1.01 [95% CI (0.66–1.55)], and the predicted odds for review score prediction for “Same country as reviewer” was 1.15 [95% CI (0.71–1.86)].

**Fig. 2:**
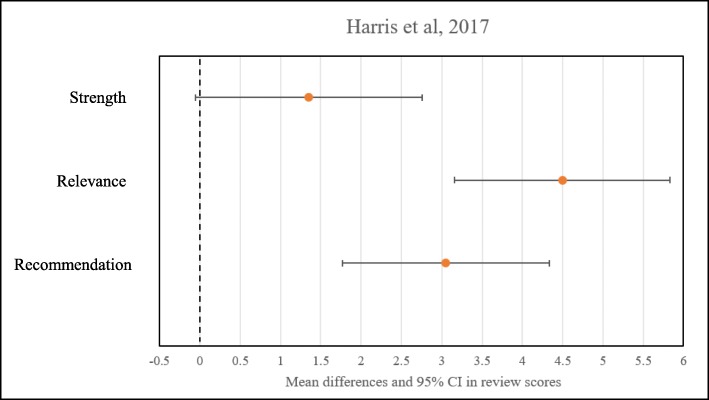
Results from Harris et al. [23]. Dotted line at 0 represents no difference in review scores. Overall mean difference in rating of strength between abstracts from HIC and LIC source was 1.35 [95% CI (− 0.06–2.76)]. Overall mean difference in rating of relevance and likelihood of recommendation to a peer between abstracts HIC and LIC source was 4.50 [95% CI (3.16–5.83)] and 3.05 [95% CI (1.77–4.33)], respectively

**Fig. 3:**
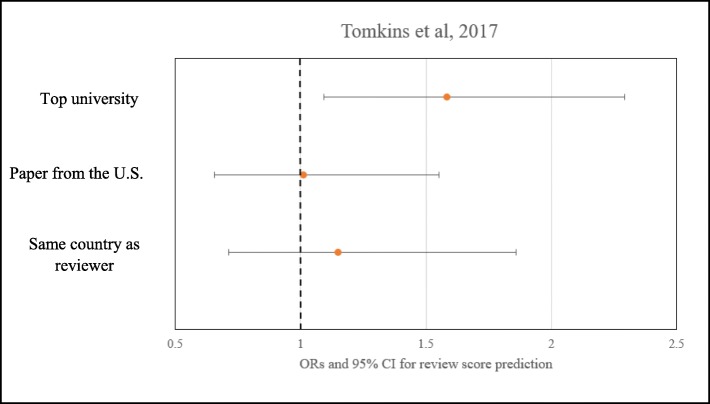
Results from Tomkins et al [24]. Dotted line at 1 represents no difference in odds of acceptance or rejection. The predicted odds for review score prediction for “Top universities” are 1.58 [95% CI (1.09–2.29]. The predicted odds for review score prediction for “Paper from the U.S.” are 1.01 [95% CI (0.66–1.55)]. The predicted odds for review score prediction for “Same country as reviewer” are 1.15 [95% CI (0.71–1.86)]

**Fig. 4:**
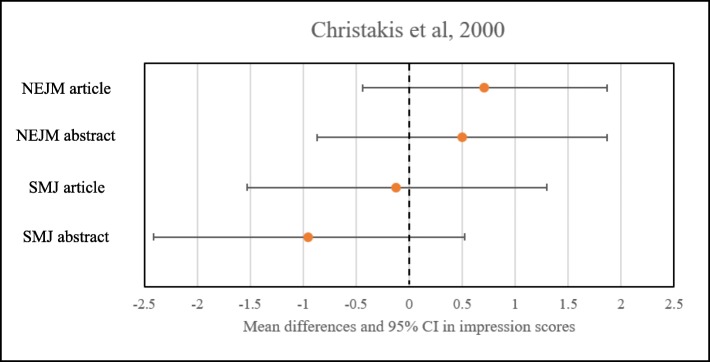
Results from Christakis et al. [25]. Dotted line at 0 represents no difference in impression scores. Mean differences in impression scores associated with attribution of an article or an abstract to the NEJM were 0.71 [95% CI (− 0.44–1.87)] and 0.50 [95% CI (− 0.87–1.87), respectively. Mean differences in impression scores associated with attribution of an article or an abstract to the SMJ were − 0.12 [95% CI (− 1.53–1.30)] and − 0.95 [95% CI (− 2.41–0.52)], respectively

Christakis [29] asked reviewers to assign scores to abstracts or articles in five categories. Each of the five characteristics was ranked on a scale of 1 to 5, with 1 as “strongly disagree” and 5 as “this is a good study.” Authors summed the responses in each category to create an aggregate “impression score” based on those five criteria. Mean differences in impression scores associated with attribution of an article or an abstract to the NEJM were 0.71 [95% CI (− 0.44–1.87)] and 0.50 [95% CI (− 0.87–1.87), respectively. Mean differences in impression scores associated with attribution of an article or an abstract to the SMJ were − 0.12 [95% CI (− 1.53–1.30)] and − 0.95 [95% CI (− 2.41–0.52)], respectively. Figures 2, 3, and 4 show summary findings for each of the included studies.

### Validity assessment

**Fig. 5:**
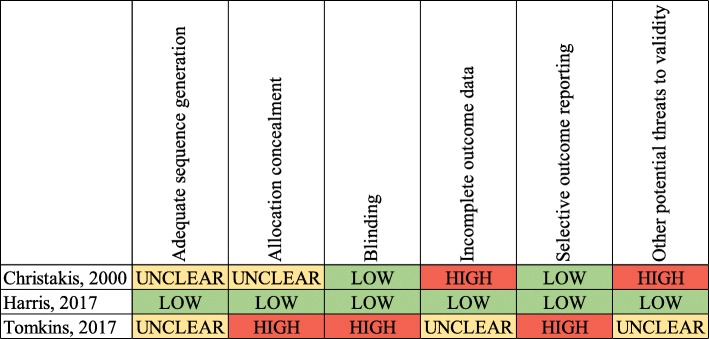
Risk of bias assessment. Risk of bias in each included study was assessed using the Cochrane Collaboration’s Risk of Bias Assessment tool [26]. Green indicates a low risk, yellow medium risk, and red high risk of bias. A more detailed discussion can be found in Additional file 1

Risk of bias in individual studies was assessed using the Cochrane Collaboration’s tool for assessing risk of bias in these three included studies [30]. A summary of this assessment can be seen in Fig. 5. An additional file shows the risk of bias assessment in more detail [see Additional file 1].

### Limitations of the included studies

As Fig. 5 shows, despite using randomization and controlled approaches, two of the included studies suffer a risk of bias. This limits the causal inferences that can be made from those studies. Further, the parallel and factorial study designs used by Tomkins and Christakis, respectively, do not provide for within-individual comparisons. Within-individual comparisons permit observations to be attributed to bias, as each individual serves as their own control. While randomization controls for confounding, neither Tomkins nor Christakis discusses in detail how randomization was carried out. Thus, we cannot conclude if observed differences in their respective results were in fact due to bias, or some other factor. Neither Christakis nor Tomkins measures whether the blinding was successful. Harris asked participants if they noticed a change in the abstracts between waves 1 and 2, and only three respondents (< 1% of participants) mentioned that they had [27], and these were accounted for in their adjusted results. While Christakis was likely able to maintain blinding throughout the study as well, Tomkins admits that participants in their study may have “unblinded” themselves in conversation with other PC members during the course of the conference [28].

## Discussion

In this systematic review, only three studies were identified to fit inclusion criteria for analysis suggesting a paucity of controlled research into the topic of geographic bias. Notwithstanding the limitations in the way the three trials we included were conducted, we found that the observation that HIC research is favored over LIC research is upheld. Therefore, on the balance of the evidence reviewed, we find that the descriptive studies have been corroborated. While descriptive studies such as the ones we cite are useful in their own right, they can only go so far in revealing explicit bias in the review and consumption of scientific literature. We find that there are few substitutes for a well-conducted, randomized, controlled crossover trial to investigate within-individual bias.

It has been largely assumed that peer review serves to improve the quality of journals [31]. But Peters and Ceci’s 1982 study was the first to call this into question [18]. Commendable progress has been made to root out some sources of bias in peer review, such as requiring the registration of clinical trials, and reporting methods for blinding and randomization [32]. However, these measures concern mostly assessment of the internal validity of the research articles. Removing information from submissions that would allow for judgments based on anything other than the quality of the research should also be strongly considered. With editors and reviewers disproportionally located in HICs, they are afforded the privileged position of “custodians” of knowledge [9, 33]. This perpetuates the uncontested knowledge hierarchy, which relegates LICs to the rank of “recipients,” rather than producers of knowledge [33, 34]. Preventing biases from manifesting by removing author affiliations or journal names from articles could prove useful. This is already done at the peer review level for many journal types through single, double, triple, and even quadruple-blinded approaches [10]. Journals like the *British Medical Journal* (BMJ) have instituted open review, where reviewers sign their reports, declare competing interests and make no further covert comments to the editors [35]. Additionally, the signed reviews are seen by the authors, along with constructively worded feedback, which can be used to resubmit a revised article [35].

Interventions should also be considered at the point of “consumption” by readers on an everyday basis. As readers harbor their own prejudices, removing information from published articles that would allow readers to judge articles based on anything other than the quality of the research should be considered. Some notable databases such as PubMed already hide author affiliation until the moment that the link is accessed and the reader is redirected to the specific journal. If geographic bias is proven to be a significant issue, then journals should explore opportunities to hide author affiliations even further to not unduly influence readers’ perceptions. The Committee on Publication Ethics (COPE) should consider developing guidance on how to address geographic bias in the peer review process, to ensure that at all stages of the publication process research is being judged based on merit alone.

Other strategies include a more decentralized, open-access, and open peer review model being employed, for instance, by F1000 [36]. Their model, which includes article submission, followed by real-time peer review and commenting on both the manuscript and the associated data, enables almost immediate, increased visibility for the research, as well as a more iterative, transparent approach to review and editing of the manuscript [36]. As pointed out by the managing director, the aim of this decentralized approach to publishing without the involvement of journals is to counteract “meaningless boundaries…that provide inappropriate and misleading metadata that is projected onto the published article,” [37]. Though journals hold a significant and valuable place in the academic community and will continue to do so, the practices employed by organizations such as F1000 may have a lasting impact on leveling the playing field between research from HICs and LICs.

Only one study [27] was able to conclusively demonstrate bias impacting on the evaluation of a research article’s relevance and one’s likelihood to recommend it to a peer, but not on the strength of the research. The two inconclusive studies [28, 29] had a weaker study design with higher risk of bias, and so their results should be interpreted with caution. Although there is descriptive evidence to suggest that geographic bias exists in research evaluation, the few RCTs investigating this subject identified through this review suggests a pressing need for further research. In addition, there is little standardization in reporting of outcome measures, making statistical comparison between studies challenging. We therefore suggest that standardization of outcome measures (such as ORs, RRs, or standardized review scores) be considered for future investigations.

Important lessons can be drawn from the included articles to support the design of future research in this space.

### Distinguishing institutional affiliation from Country of Origin

**Fig. 6:**
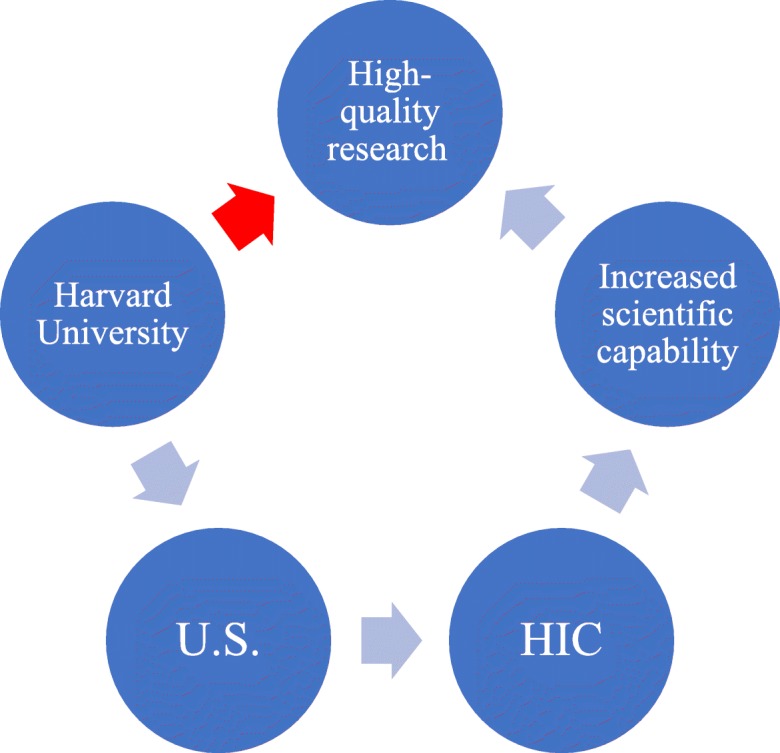
Heuristic framework. Reviewers may see “Harvard University,” and through a series of reasonable assumptions arrive at the conclusion that Harvard produces high-quality research (blue arrows). The heuristic occurs when reviewers see “Harvard University” and necessarily assume that the research is of high quality, when this may not be the case

To a greater or lesser extent, COO effects are elicited by the institution name. It is reasonable to presume that high-quality research necessitates a certain level of economic development [8], and if a university will be associated to a country, and that country will be associated with a level of economic development, this in turn will imply a certain amount of scientific capability, and the possibility for producing high-quality research. Often, it is clear whether the institution can be associated to a particular country. For example, Harris et al [27] used “University of Addis Ababa, Ethiopia” as one of the LIC institutional sources for the abstracts in their study, and so the COO cue is clear, not just because the country is cited, but because Addis Ababa is clearly the capital of Ethiopia. However, they also used “Harvard University, U.S.” and although it is clear that the U.S. is the COO cue, “Harvard University” has such a strong brand recognition that for most readers it would be clear that even if used alone it would be referring to the U.S. However, had an institution been used that neither has strong brand recognition, nor obvious geographic affiliation, then the external country cue might not be as clear, and the extent to which any elicited bias was due to geographic bias would have been uncertain. Thus, even if Harris et al. [27] had not also indicated the COO (“Ethiopia” or “U.S.”, respectively) in their study, reviewers may have automatically associated “Harvard University” with high-quality research (Fig. 6) or linked “University of Addis Ababa” to “LIC,” a lower degree of scientific capability, and poor-quality research.

Using just institutional affiliations can be sufficient to link a given research article to a specific country, thus eliciting the geographic bias where it is present, but some care must be taken when doing so in controlled studies. Tomkins [28] found that when reviewers were aware that the article under review originated from a top university, they were more likely to recommend it for acceptance. Although they do not explicitly draw the connection between university ranking and the country’s income status, 45 of the 50 top-ranked universities in Computer Science and Information Systems are in HICs [38], and so reviewers may have been considering country income status rather than, or as well as, institutional prestige. In other words, reviewers may have been basing their recommendation to accept a given manuscript on the COO of that manuscript, implicitly favoring those submissions from top-ranked institutions in HICs. Future controlled studies using factorial designs will be able to distinguish between the relative importance of an institution brand and country brand for eliciting the COO cue.

### Effect of journal attribution

Scientific journals can be viewed as products, and as with most other products, they may elicit some geographic stimulus. Particularly if their names involve an explicit geographic identifier, they may be evaluated differently based on their COO [17]. As such, renowned journals such as the NEJM, the *Journal of the American Medical Association* (JAMA), or the BMJ, which originate from HICs, could lead readers to assume that they are reading “high-quality” research by virtue of the fact that they are implicitly associated with HICs. Conversely, less recognizable journals, such as the SMJ, or the *African Journal of Environmental Sciences and Technology* (AJEST) may not benefit from this treatment and may even be evaluated less favorably because of their geographic origin.

The role of journal attribution per se was investigated by two studies [27, 28]. Harris and colleagues [27] concluded that there was no significant effect of the interaction between journal type (high or low IF) and country source and that changing the country source was more significant than changing the journal type. Similarly, Christakis and colleagues [29] found journal attribution played no statistically significant role in impression scores between attributed and unattributed articles and abstracts, when adjusting for other covariates. Nonetheless, studies exploring geographic bias in research evaluation need to take into account the listed journal type and whether any geographic identifier is present. The NEJM and the BMJ both have strong country cues. *The Lancet* has strong brand recognition as a U.K.-based publication. F1000, however, is an international consortium without a specific geographic identifier. Future controlled studies should examine the relative importance of the journal characteristics in eliciting COO cues and geographic bias.

Although geographic bias may not be restricted only to the axis of HICs versus LICs and it may occur at local, regional, and national levels, it is likely that LICs are most affected by elicited biases. In the humanities and social sciences and increasingly in the biomedical sciences, some academic institutions in HICs are beginning to re-evaluate their curricula to challenge predominantly western narratives and include more diverse voices and bodies of thought [39]. Such initiatives aim to bring non-western narratives and experiences to the fore and interrupt the continuous feedback of western superiority which is the basis to this sort of geographic bias [40].

Improving visibility of LIC research through scientific collaboration is now easier than ever before through open access publication and research collaboration [6]. However, paying for fees associated with open access publishing, and remunerating authors for their expenses, may remain a privilege enjoyed by those affiliated with institutions in HICs, thus creating another barrier to parity in publication. Collaboration can be particularly important to LICs, as it introduces new technologies and capabilities which allow for research and development to continue in the future [8], although care must be taken to ensure equitable recognition which is still predominantly benefitting researchers from the HICs [4]. Considering the ties between a nation’s scientific capability and its economic progress [8], developing research partnerships could prove to be the best way to participate in the scientific discourse [41]. In the medium term, this will empower countries through mutually beneficial partnerships [41]. A more long-term objective should be the creation of sustainable policies surrounding international development, which must include a strong focus on capacity-building and scientific collaboration between HICs and LICs/LMICs.

### Limitations

This review does not comprise the universe of published literature regarding COO effects and geographic bias because our search, whilst systematic and comprehensive, involved only five major databases, and although we do not have reason to believe there will be other unidentified studies, we cannot exclude that possibility. Considering the large amount of titles retrieved (3501), a pragmatic decision was made to have only one reviewer screen retrieved titles, rather than two, as would be standard practice. This may also have led to relevant studies being missed. We settled on the inclusion criteria that we chose in an effort to identify only the most robustly conducted studies, using peer-reviewed, controlled, and randomized methods, so that comment could be reliably made on the role that explicit geographic bias plays in research review. The inevitable trade-off between breadth and specificity certainly played out in this research question, and widening the search and inclusion criteria could expand the selection of articles included. Future investigations could be expanded to include abstracts submitted to conferences, such as the Peer Review Congress and the Cochrane Colloquium.

Relying solely on articles published in English likely also resulted in additional relevant articles being overlooked. A further, more exhaustive review across multiple fields, and in several languages, is warranted. Earlier iterations of the search terms were more detailed and complex than the ones ultimately used; however, more complex combinations yielded fewer results, potentially excluding relevant articles. Therefore, a more simplistic search strategy was used, relying on screening and manual exclusion of irrelevant articles. This ensured that pertinent articles would not be inadvertently excluded by the search strategy. Nonetheless, it is possible that this simpler search strategy did not include some important keywords and subject headings. This may have led us to overlook other relevant research on the topic.

We used the Cochrane Collaboration’s tool for measuring the risk of bias because this tool is most applicable for the assessment of bias in randomized trials. However, as our focus was on non-clinical trials, the methods used in the studies included in this review may differ from the methods employed in clinical settings and as such, given the context of this paper, the tool may not be as applicable to the assessment of bias and may not appropriately reflect the true risk of bias. Nonetheless, we did find that only one of three included studies had a low risk of bias.

## Conclusion

This systematic review identified three RCTs that investigate the role of geographic bias in research evaluation and peer review. There is strong evidence provided by one robust experimental study on the topic, suggesting evidence of geographic bias in the evaluation of medical research by English clinicians, but the methodological variety and risk of bias in the remaining studies retrieved make it challenging to draw firm conclusions regarding the extent to which geographic bias elicited from institutional affiliation or COO of authors impacts on the evaluation of research more broadly. Further RCTs are necessary to conclusively determine the effect that COO has on the evaluation of scientific research. At present, the call to address inequalities in knowledge production and publication has never been greater. By drawing attention to the role geographic bias plays in the process of knowledge diffusion, prejudice against LIC research, but also other forms of geographic bias, can be addressed and rooted out among the reviewers and editors of scientific publications, and among those who read, cite, and consume those scientific publications. Indeed, academics, editors, and journal editorial boards all have important roles to play in addressing this issue.

## Supplementary information

**Additional file 1.** Detailed risk of bias assessment. Description of data: A more detailed discussion of the risk of bias assessment of the included studies, which resulted in the abbreviated version seen in Fig. 5.

## Data Availability

The datasets used and/or analyzed during the current study are available from the corresponding author on reasonable request.

## Abbreviations

ACE: Angiotensin-converting enzyme
AJEST: African Journal of Environmental Sciences and Technology
AMA: American Medical Association
BMJ: British Medical Journal
CI: Confidence interval
COO: Country of origin
COPE: Committee on Publication Ethics
DBPC: Double-Blind Program Committee
DOTS: Directly observed treatment, short course
GDP: Gross domestic product
HIC: High-income country
HIV: Human immunodeficiency virus
HMIC: Health Management Information Consortium
IF: Impact factor
JAMA: Journal of the American Medical Association
LIC: Low-income country
LMIC: Lower-middle-income country
NEJM: New England Journal of Medicine
NSAID: Nonsteroidal anti-inflammatory drug
OR: Odds ratio
PC: Program Committee
RCT: Randomized controlled trial
RR: Relative risk
RWJ: Robert Wood Johnson
SBPC: Single-Blind Program Committee
SMJ: Southern Medical Journal
TB: Tuberculosis
WSDM: Web Science and Data Mining
